# The effects of selective head cooling versus whole-body cooling on some neural and inflammatory biomarkers: a randomized controlled pilot study

**DOI:** 10.1186/s13052-015-0188-5

**Published:** 2015-10-15

**Authors:** Yalçın Çelik, Aytuğ Atıcı, Selvi Gülaşı, Khatuna Makharoblıdze, Gülçin Eskandari, Mehmet Ali Sungur, Serin Akbayır

**Affiliations:** Mersin University School of Medicine, Department of Neonatology Care Unit, 33343 Mersin, Turkey; Mersin University Faculty of Medicine, Department of Neonatology, 33343 Mersin, Turkey; Mersin University School of Medicine, Department of Neonatology, 33343 Mersin, Turkey; Mersin University Faculty of Medicine, Department of Pediatric Neurology, 33343 Mersin, Turkey; Mersin University Faculty of Medicine, Department of Biochemistry, 33343 Mersin, Turkey; Mersin University Faculty of Medicine, Department of Biostatistics, 33343 Mersin, Turkey

**Keywords:** Biomarkers, Hypoxic ischemic encephalopathy, Selective head cooling, Whole body cooling

## Abstract

**Background:**

Therapeutic hypothermia (TH) has become standard care in newborns with moderate to severe hypoxic ischemic encephalopathy (HIE), and the 2 most commonly used methods are selective head cooling (SHC) and whole body cooling (WBC). This study aimed to determine if the effects of the 2 methods on some neural and inflammatory biomarkers differ.

**Materials and methods:**

This prospective randomized pilot study included newborns delivered after >36 weeks of gestation. SHC or WBC was administered randomly to newborns with moderate to severe HIE that were prescribed TH. The serum interleukin (IL)-1β, IL-6, neuron-specific enolase (NSE), brain-specific creatine kinase (CK-BB), tumor necrosis factor-alpha (TNF-α), and protein S100 levels, the urine S100B level, and the urine lactate/creatinine (L/C) ratio were evaluated 6 and 72 h after birth. The Bayley Scales of Infant and Toddler Development-III was administered at month 12 for assessment of neurodevelopmental findings.

**Results:**

The SHC group included 14 newborns, the WBC group included 10, the mild HIE group included 7, and the control group included 9. All the biomarker levels in the SHC and WBC groups at 6 and 72 h were similar, and all the changes in the biomarker levels between 6 and 72 h were similar in both groups. The serum IL-6 and protein S100 levels at 6 h in the SHC and WBC groups were significantly higher than in the control group. The urine L/C ratio at 6 h in the SHC and WBC groups was significantly higher than in the mild HIE and control groups. The IL-6 level and L/C ratio at 6 and 72 h in the patients that had died or had disability at month 12 were significantly higher than in the patients without disability at month 12.

**Conclusion:**

The effects of SHC and WBC on the biomarkers evaluated did not differ. The urine L/C ratio might be useful for differentiating newborns with moderate and severe HIE from those with mild HIE. Furthermore, the serum IL-6 level and the L/C ratio might be useful for predicting disability and mortality in newborns with HIE.

## Background

Worldwide, hypoxic ischemic encephalopathy (HIE) is a primary cause of disability and mortality in newborns [1–7]. Currently, therapeutic hypothermia (TH) is routinely used to treat moderate and severe HIE [8–13]. TH is administered as selective head cooling (SHC) or whole-body cooling (WBC), but it is not known if the efficacy of these 2 methods differ [14–19]. Regardless of which method is used, it is recommended that TH be initiated for HIE as early as possible, preferably within 6 h of birth [9]. The diagnosis of HIE and making the decision to start TH can be difficult. Furthermore, TH is not effective in all cases of HIE and despite administration of TH severe disability can occur [1, 20]. As such, researchers have been trying to identify a biomarker that can be used to diagnose HIE, to determine if TH should be administered, and to predict prognosis [21–23].

Earlier studies have reported some promising biomarkers [21]. A recent study reported that TH administered using WBC suppressed cytokine levels in newborns with HIE, which was suggested to play a role in the neuroprotective effect of TH [8]. This finding suggests that SHC and WBC may have different effects on inflammatory and neuronal biomarkers. To the best of our knowledge, no study has compared the effect of SHC and WBC on biomarkers; therefore, the present study aimed to determine if the effects of these 2 methods on some neural and inflammatory biomarkers differ. An additional aim was to determine if the studied biomarkers are useful for predicting disability and mortality in newborns with HIE.

## Methods

This prospective randomized study included newborns born after >36 weeks of gestation that were inpatients at Mersin University, School of Medicine, Neonatal Intensive Care Unit, Mersin, Turkey, between April 2010 and December 2011. The study protocol was approved by the Mersin University Ethics Committee and written informed consent was obtain from the families of the newborns. During randomization, age >6 h, severe intrauterine growth retardation, and congenial anomaly were considered exclusion criteria. HIE was diagnosed according to American College of Obstetricians and Gynecologists criteria [24], and modified Sarnat staging was used to classify HIE [25]. Passive cooling (turning off the incubator heater during transfer) was administered to newborns with HIE that were transferred from other hospitals.

The decision to administer TH to the newborns with HIE was based on having ≥1 of criteria A, B, and C, as shown in Table 1. SHC or WBC was selected randomly via the closed envelope method. SHC was administered using a manually controlled device (Olympic Medical Cool Care System, Olympic Medical, Seattle, WA, USA), whereas WBC was administered using a room air conditioning system. Body temperature was continuously monitored via rectal probes in the SHC and WBC groups. In both groups rectal temperature was recorded every 30 min. The targeted 72-h rectal temperature for WBC was 33–34 °C, versus 34–35 °C for SHC. After the initial 72 h the rectal temperature was increased for both TH methods to 36.5 °C via increasing the temperature 0.5 °C h^−1^. Patients that were not in the targeted temperature range for >1 h were excluded.

**Table 1:** Criteria indicative for therapeutic hypothermia

Criteria A
1. Apgar score <5 at 10 min of age
2. Continued need for ventilation 10 min after birth
3. pH <7.0 or base deficit < −16 in blood gases within 1 h of birth
Criteria B
Moderate to severe encephalopathy, consisting of altered state of consciousness (as shown by lethargy, stupor, or coma) and >1 of the following:
1. Hypotonia
2. Abnormal reflexes, including oculomotor or papillary abnormalities
3. Absent or weak sucking reflex
4. Clinical seizures
Criteria C
Amplitude-integrated electroencephalography (aEEG) records (30-min)
1. Moderately abnormal aEEG background activity
2. Severely abnormal aEEG background activity
3. Seizure activity

Amplitude-integrated electroencephalography (aEEG) findings were defined as severely abnormal based on continuous low voltage, burst suppression, or flat tracing in the ground pattern, versus moderately abnormal based on discontinuous normal voltage [26–28]. Newborns diagnosed as HIE that did not meet all the criteria for TH constituted the mild HIE group. Newborns in the neonatal intensive care unit with transient tachypnea, an Apgar score >7 at 1 and 5 min, blood gas pH >7.30 within 1 h after birth, and didn’t have clinical or culture positivity for sepsis constituted the control group.

From all the included newborns 2 mL of blood and 2 mL of urine were collected 6 and 72 h after birth. The blood samples were centrifuged, and then the separated serum was stored at −80 °C until analyzed for interleukin (IL)-6, IL-1β, tumor necrosis factor-alpha (TNF-α), brain-specific creatine kinase (CK-BB), protein S100B via enzyme-linked immunosorbent assay, and neuron-specific enolase (NSE) and protein S100 via enzyme immunoradiometric assay. Urine lactate and creatinine levels were measured in fresh urine samples. Lactate was measured via the colorimetric lactate oxidase method and creatinine was measured via the enzymatic creatinine method, using a Cobas Integra 800 analyzer (Roche Diagnostics).

The newborns were evaluated at age 12 months for disability, neuromotor retardation, and mortality. Cognitive, language, and motor development at 12 months were evaluated using the Bayley Scales for Infant and Toddler Development-III (BSID- III) [29]. Those with a cognitive, language, or motor combined score 2 standard deviations lower than the mean BSID-III score, total loss of vision, or cerebral palsy were considered severely disabled. Those with a cognitive, language, or motor combined score 1 standard deviation lower than the BSID-III mean were defined as neuromotor retardation. Those with normal neurological examination findings without audio or visual problems, and those with a cognitive, language, and motor combined BSID-III score >85 were considered disability free.

### Statistical analysis

Statistical analysis was performed using PASW v.18.0 (Predictive Analytics Software, a registered trademark of SPSS, Inc.) and Statistica v.8.0 (demo version). The distribution of continuous variables was evaluated using the Shapiro-Wilks test. Continuous variables with normal distribution were compared between groups using one-way ANOVA and the Scheffe post hoc test for multiple comparisons was used to determine difference between groups. Continuous variables not normally distributed were compared between groups using the Kruskal-Wallis test and Dunn test. Findings at the 12-month evaluation were compared between groups using the Mann–Whitney *U* test. The Wilcoxon signed-rank test was used to compare findings at 6 and 72 h within groups. The Fisher-Freeman exact test was used when the expected value rule wasn’t fulfilled, and the chi-square was used to evaluate the relationships between the categorical variables. Continuous variables are shown as mean ± SD or median (IQR), depending on the distribution. The level of statistical significance was set at *P* < 0.05.

## Results

During the study period, 54 newborns were diagnosed as HIE, of which 11 were excluded because they were aged >6 h at the time of randomization, 4 were excluded because died before 6 h, and 1 was excluded due to a congenital malformation. Ten newborns not meeting criteria for TH were included in the mild HIE group. Of 28 infants meeting criteria for TH, 15 were randomly allocated into group receiving SHC and 13 were allocated into group receiving WBC. One of the newborns in the WBC group was later excluded from the study because of the development of treatment-resistant hypotension that resulted in discontinue of the cooling therapy. In addition, serum and urine samples were not timely collected in 1 patient in the SHC group, 2 patients in the WBC group, 3 patients in the mild HIE group, and 1 case in the control group. After that the SHC group included 14 patients, the WBC group included 10 patients, the mild HIE group included 7 patients and the control group included 9 patients. The targeted rectal temperature was maintained for 72 h in all the patients in the SHC and WBC groups. Baseline patient characteristics in the SHC and WBC groups were similar (*p* > 0.05). Baseline patient characteristics in all groups are shown in Table 2, and biomarker levels at 6 and 72 h are given in Tables 3 and 4. The IL-6 level at 6 h was similar in the SHC and WBC groups (*P* > 0.05), and was significantly higher than in the control group (*P* = 0.026 for SHC- control and *P* = 0.015 for WBC- control). The serum protein S100 level at 6 h was similar in the SHC and WBC groups (*P* > 0.05), and was significantly higher than in the control group (*P* = 0.013 for SHC- control and *P* = 0.031 for WBC- control). The L/C ratio at 6 h was similar in the SHC and WBC groups (*P* > 0.05), and was significantly higher than in the mild HIE group (*P* = 0.008 for SHC- mild HIE and *P* = 0.014 for WBC- mild HIE) and control group (*P* = 0.001 for SHC- control and *P* = 0.002 WBC- control). The levels of all the other biomarkers (serum IL-1β, TNF-α, NSE, CKBB, and urine S100B) at 6 h were similar in each group (*P* > 0.05). The IL-6 level at 72 h was similar in the SHC and WBC groups (*P* > 0.05), and was significantly higher than in the mild HIE group (*P* = 0.004 for SHC- mild HIE and *P* = 0.003 for WBC- mild HIE) and control group (*P* = 0.018 for SHC- control and *P* = 0.014 for WBC- control). The levels of all other biomarkers at 72 h were similar in each group (*P* > 0.05).

**Table 2:** Baseline patient characteristics

	SHC (*n* = 14)	WBC (*n* = 10)	Mild HIE (*n* = 7)	Control (*n* = 9)	P
Gestational age (weeks)	38.6 ± 1.1	38.9 ± 1.0	38.8 ± 1.1	38.8 ± 0.8	0.931
Birth weight (g)	3183 ± 501	3218 ± 445	3410 ± 306	3081 ± 289	0.480
Head circumference (cm)	34.7 ± 1.1	34.7 ± 0.7	34.5 ± 1.0	34.6 ± 0.6	0.945
Male	4 (28.6)	3 (30.0)	2 (28.6)	4 (44.4)	0.905
Delivery via cesarean section	2 (14.3)	4 (40.0)	2 (28.6)	3 (33.3)	0.512
Birth in the treatment center	1 (7.1) ^a^	0 (0.0) ^a^	0 (0.0) ^a^	7 (77.8) ^b^	<0.001
Age at group randomization (h)	5.4 ± 1.6	5.9 ± 0.3	5.3 ± 1.1	4.6 ± 1.9	0.225
Rectal temperature at group randomization (°C)	34.1 ± 0.9	34.4 ± 1.3	NA	NA	0.615
Existence of clinical convulsions	13 (92.9) ^a^	9 (90.0) ^a^	0 (0.0) ^b^	0 (0.0) ^b^	<0.001
Invasive mechanical ventilation	13 (92.9) ^a^	9 (90.0) ^a^	2 (28.6) ^b^	0 (0.0) ^b^	<0.001
5-min Apgar score (*n* = 35)					
0–3	5 (50.0)	4 (40.0)	0 (0.0)	0 (0.0)	
4–6	5 (50.0)	6 (60.0)	5 (83.3)	0 (0.0)	
7–10	0 (0.0)	0 (0.0)	1 (16.7)	9 (100)	
10-min Apgar score (*n* = 24)					
0–3	1 (14.3)	3 (75.0)	0 (0.0)	0 (0.0)	
4–6	6 (85.7)	1 (25.0)	1 (25.0)	0 (0.0)	
7–10	0 (0.0)	0 (0.0)	3 (75)	9 (100)	
Blood gas analysis within 1 h post birth					
pH	6.9 ± 0.2^a^	7.0 ± 0.1^a^	7.0 ± 0.1^a^	7.30 ± 0.1^b^	0.001
Base excess (mmol L^−1^)	−17.7 ± 2.9^a^	−19.6 ± 1.2^a^	−17.8 ± 8.9^a^	−2.6 ± 1.3^b^	<0.001
aEEG before randomization					
Normal	0 (0.0)	0 (0.0)	7 (100)	NA	<0.001
Moderate abnormality	3 (21.4)	1 (10.0)	0 (0.0)	NA	
Severe abnormality	11 (78.6)	9 (90.0)	0 (0.0)	NA	
Sarnat stage					
Stage I	0 (0.0)	0 (0.0)	7 (100)	NA	<0.001
Stage II	3 (21.4)	1 (10.0)	0 (0.0)	NA	
Stage III	11 (78.6)	9 (90.0)	0 (0.0)	NA	

Values are given as median ± SD, or number of patients and percentage

^a^ and ^b^ denote differences between groups, according to post hoc test. There is a significant difference data shown with different letters (*P* < 0.05)

**Table 3:** Biomarker levels at 6 h

Biomarkers	SHC group (*n* = 14)	WBC group (*n* = 10)	Mild HIE group (*n* = 7)	Control group (*n* = 9)
IL-6 (pg mL^−1^)	49.5^a^ (15.8–219.9)	46.7^a^ (33.1–413.5)	24.7^a,b^ (14.0–69.6)	3.8^b^ (2.8–6.6)
IL-1β (pg mL^−1^)	2.2 (2.1–2.3)	2.5 (2.1–3.0)	2.1 (2.0–2.6)	2.1 (2.0–2.2)
TNF-α (pg mL^−1^)	15.2 (10.8–25.6)	15.8 (13.7–41.6)	20.3 (13.9–25.6)	15.3 (13.1–24.4)
NSE (μg L^−1^)	0.58 (0.44–1.62)	0.67 (0.48–2.65)	0.49 (0.37–2.13)	0.45 (0.36–0.51)
S100B (ng mL^−1^)	0.08 (0.07–0.09)	0.12 (0.07–0.15)	0.11 (0.08–0.15)	0.07 (0.06–0.08)
S100 (ng L^−1^)	17.5^a^ (5.6–60.4)	15.3^a^ (7.7–86.9)	1.7^a,b^ (0.8–25.6)	1.9^b^ (0.7–4.7)
CKBB (ng mL^−1^)	1.9 (1.5–3.0)	2.1 (1.4–3.9)	1.9 (1.4–2.3)	1.4 (1.1–1.8)
L/C ratio	9.24^a^ (1.32–43.89)	3.00^a^ (0.82–34.60)	0.05^b^ (0.03–0.11)	0.04^b^ (0.03–0.08)

Data shown as median (IQR)

^a^ and ^b^ denote differences between groups, according to post hoc test. There is a significant difference data shown with different letters (*P* < 0.05)

**Table 4:** Biomarker levels at 72 h

Biomarkers	SHC group (*n* = 14)	WBC group (*n* = 10)	Mild HIE group (*n* = 7)	Control group (*n* = 9)
IL-6 (pg mL^−1^)	64.2^a^ (30.4–118.0)	43.0^a^ (33.1–416.0)	4.3^b^ (1.2–17.6)	3.0^b^ (1.3–6.0)
IL-1β (pg mL^−1^)	2.2 (2.0–2.3)	2.2 (2.1–2.6)	2.3 (2.1–2.7)	2.1 (2.0–2.2)
TNF-α (pg mL^−1^)	19.5 (12.5–41.7)	17.0 (13.3–25.3)	24.3 (20.2–40.6)	15.7 (10.8–16.7)
NSE (μg L^−1^)	0.51 (0.38–0.74)	0.57 (0.38–1.70)	1.01 (0.40–1.93)	0.38 (0.32–0.47)
S100B (ng/ml	0.08 (0.07–0.15)	0.10 (0.06–0.15)	0.12 (0.07–0.21)	0.08 (0.07–0.12)
S100 (ng L^−1^)	2.9 (0.5–15.5)	9.5 (0.3–29.8)	1.6 (0.8–3.6)	1.5 (0.4–3.8)
CKBB (ng mL^−1^)	1.0 (0.6–1.7)	0.8 (0.5–1.5)	0.9 (0.7–1.1)	1.0 (0.8–1.1)
L/C ratio	0.08 (0.04–0.27)	0.06 (0.04–0.16)	0.05 (0.03–0.06)	0.03 (0.02–0.04)

Data shown as median (IQR)

^a^ and ^b^ denote differences between groups, according to post hoc test. There is a significant difference data shown with different letters (*P* < 0.05)

The IL-6 level decreased significantly from 6 to 72 h only in the mild HIE group (*P* = 0.028). The CK-BB level decreased significantly from 6 to 72 h in all groups (*P* < 0.05) and the L/C ratio decreased significantly from 6 to 72 h in the SHC (*P* = 0.008), WBC (*P* = 0.018), and control (*P* = 0.028) groups. The other biomarker levels did not change significantly between 6 and 72 h in any of the groups. Additionally, the difference between 6 and 72 h for all the biomarkers was calculated in the SHC and WBC groups in order to determine if the effect of the 2 methods differed; the increase or decrease in each biomarker between 6 and 72 h was determined in percentages and there weren’t any significant differences between the 2 groups (*P* > 0.05).

At the age 12 months evaluation in the SHC group (*n* = 14) 5 of the patients had died, 5 were severely disabled, 1 had neuromotor retardation, and 3 were disability free, whereas in the WBC group (*n* = 10) 5 of the patients had died, 2 were severely disabled, and 3 were disability free. At the age 12 months evaluation all 7 of the patients in the mild HIE group were alive and disability free. Among the 31 HIE patients in the study, at age 12 months 18 were adverse outcomes (died, severe disability, or neuromotor retardation) and 13 were favorable outcomes (living without disability). The serum IL-6 level and urine L/C ratio at 6 and 72 h were significantly higher in the patients that had adverse outcomes than in the patients that had favorable outcomes at 12 months (IL-6: *P* = 0.049 and *P* = 0.013, respectively; L/C ratio: *P* < 0.001 and *P* = 0.027, respectively). There weren’t any significant relationships between the other 6th or 72nd hour biomarker levels and 12th month outcomes (Tables 5 and 6).

**Table 5:** The relationship between biomarker levels at 6 h and outcomes at age 12 months

Biomarkers	Patients with adverse outcomes (*n* = 18)	Patients with favorable outcomes (*n* = 13)
IL-6 (pg mL^−1^)	121.1 (31.4–334.3)*	33.2 (13.0–61.7)
IL-1β (pg mL^−1^)	2.3 (2.1–2.5)	2.3 (2.0–2.5)
TNF-α (pg mL^−1^)	16.6 (10.8–32.7)	16.5 (13.3–21.0)
NSE (μg L^−1^)	0.55 (0.44–1.10)	1.31 (0.46–2.15)
S100B (ng mL^−1^)	0.08 (0.06–0.13)	0.08 (0.07–0.15)
S100 (ng L^−1^)	13.5 (5.8–31.0)	22.4 (1.3–136.4)
CKBB (ng mL^−1^)	2.1 (1.5–3.5)	1.9 (1.4–2.4)
L/C ratio	19.0 (2.64–47.89)*	0.12 (0.04–0.36)

Data shown as median (IQR)

*There is a significant difference between groups (*P* < 0.05)

**Table 6:** The relationship between biomarker levels at 72 h and outcomes at age 12 months

Biomarkers	Patients with adverse outcomes (*n* = 18)	Patients with favorable outcomes (*n* = 13)
IL-6 (pg mL^−1^)	60.3 (29.1–121.1)*	24.6 (3.9–45.0)
IL-1β (pg mL^−1^)	2.1 (2.0–2.2)	2.3 (2.1–2.7)
TNF-α (pg mL^−1^)	17.7 (12.2–41.3)	22.8 (15.7–31.9)
NSE (μg L^−1^)	0.52 (0.38–0.74)	0.98 (0.44–2.13)
S100B (ng mL^−1^)	0.09 (0.06–0.12)	0.11 (0.07–0.21)
S100 (ng L^−1^)	1.9 (0.4–15.8)	3.5 (0.7–20.1)
CKBB (ng mL^−1^)	0.8 (0.6–1.5)	1.0 (0.7–1.3)
L/C ratio	0.13 (0.04–0.26)*	0.04 (0.03–0.06)

Data shown as median (IQR)

*There is a significant difference between groups (*P* < 0.05)

## Discussion

In the present study there weren’t any differences between the effects of SHC and WBC on the evaluated biomarkers in newborns with moderate and severe HIE. The IL-6 level and L/C ratio at 6 and 72 h in the patients that had died, had severe disability, and had neuromotor retardation at the time of the 12-month evaluation were significantly higher than in those that were alive without disability at month 12.

Although the precise mechanism of cellular damage caused by hypoxia-ischemia remains unknown, energy deficiency, excitatory neurotransmitters and free oxygen radicals are considered to contribute to the cascade of events leading to injury [30]. In addition, inflammation is known to play an important role in the pathogenesis of HIE [30]. Hypoxia-ischemia is followed by cerebral and peripheral immune response. Microglia and astrocytes become activated on one hand, while peripheral monocytes infiltrate the brain due to disrupted blood–brain barrier on the other [8]. Activated inflammatory cells release pro-inflammatory cytokines and chemokines such as IL-1, IL-2, IL-6, IL-8, TNF-α, and interferons [8, 30]. As a result, inflammatory biomarkers’ levels increase in HIE. On the other hand, various intracellular biomarkers like glial fibrillary acidic protein, NSE, and Protein S100 shift to extracellular compartment due to cell damage during HIE [31].

Researchers are trying to identify a biomarker suitable for diagnosing HIE, and for predicting the severity of disease and prognosis in the early period. Many biomarkers in blood, urine, and cerebrospinal fluid (CSF) have been studied, some of which were reported to be promising [21, 23]. The present study analyzed biomarkers that had been previously studied and were reported to be promising.

Nagdyman et al. [32] studied CK-BB, protein S100, and NSE levels in cord blood and serum collected at 2, 6, 12, and 24 h post birth in 7 newborns with moderate and severe HIE, and in 22 with mild HIE. The protein S100 and CK-BB levels in all the newborns with HIE at all time points were significantly higher than in the control group. In addition, the serum protein S100 and CK-BB levels at 2 and 6 h were significantly higher in the newborns with moderate and severe HIE than in those with mild HIE. There wasn’t a significant difference in the serum NSE level between the newborns with HIE and the control group at any time point. Similarly, in the present there wasn’t a significant difference in the serum NSE level between the newborns with HIE and the control group. Additionally, the protein S100 level at 6 h was significantly higher in the newborns with moderate and severe HIE (SHC and WBC) than in the control group. Moreover, in contrast to Nagdyman et al.’s [30] study there wasn’t a significant difference in the serum CK-BB level between the present study’s newborns with and without HIE, which might be because TH was administered in the present study’s newborns with moderate and severe HIE, but not in Nagdyman et al.’s patients.

Gazzolo et al. [33] studied the urine S100B level at 0, 24, 48, and 96 h in newborns with HIE and healthy controls. They reported that the urine S100B level in the newborns with HIE that died at age 1–7 d (*n* = 12) was significantly higher than in those that were alive on d 7 (*n* = 48) and the control group (*n* = 72). In the present study the urine S100B level was similar in the SHC, WBC, mild HIE, and control groups, and a non-significant correlation was observed between the adverse outcomes at month 12, and the S100B level at 6 and 72 h. The difference in findings between the present study and Gazzolo et al.’s might be due to administration of TH in the present study. Moreover, an earlier study [34] reported that the S100B level was significantly lower in newborns with HIE treated with TH than in newborns with similar properties with HIE that weren’t treated with TH.

Huang et al. [35] studied the urine L/C ratio at 6 and 48–72 h in 40 newborns with HIE and 58 healthy controls. They observed that the urine L/C ratio was significantly higher in the newborns with HIE and the mean ratio of urinary L/C was significantly higher in the infants who had adverse outcomes (death, severe cerebral palsy, BSID II score that was more than 2 SD below the mean score for age, blindness, or deafness) at month 12 than the infants with favorable outcomes (normal neurologic development or slight abnormalities in muscle tone and reflexes). A similar study by Ghotbi et al. [36] reported that the L/C ratio at 6 and 24 h was significantly higher in 50 newborns with HIE than in 50 healthy controls. In the present study TH was administered in newborns with moderate and severe HIE, in contrast to the other studies, and the L/C ratio was observed to be significantly higher in the newborns with moderate and severe HIE than in those in the mild HIE and control groups. Furthermore, the L/C ratio was higher at 6 and 72 h in the present study’s patients that had died, had severe disability, and had neuromotor retardation at month 12 than in those that were living without disability, as reported by Huang et al. [35]. These findings suggest that the urine L/C ratio might be useful for identifying newborns with moderate and severe HIE, and for predicting disability and mortality.

Massaro et al. [9] studied the serum NSE and S100B levels at 0, 12, 24, and 72 h of hypothermia in newborns with HIE that received TH using WBC and their relationship to neurodevelopmental results. They reported that there was a significant relationship between serum NSE and S100B levels, and neurodevelopmental results at month 15. In the present study there wasn’t a significant relationship between adverse outcomes month 12, and NSE or S100B levels at 6 and 72 h. It is possible that these different results may be due to the heterogeneity of the etiologies that lead to HIE, timing of the insult and variability of the severity.

Chalak et al. [20] administered WBC in 20 newborns with moderate and severe HIE, but not in 7 with mild HIE. They analyzed various neuronal and inflammatory biomarkers in the blood samples obtained at 6–24, 48, 72, and 78 h. They reported that the IL-6 level at 6–24 h was significantly higher in the newborns with moderate and severe HIE than in those with mild HIE. Furthermore, they noted that there was a significant relationship between the IL-6 and TNF-α levels at 6–24 h, and abnormal neurological results at 15–18 months. In the present study the IL-6 level at 6 h was significantly higher in the SHC and WBC groups than in the control group, and the IL-6 level at 72 h was significantly higher in the SHC and WBC groups than in the mild HIE and control groups. As reported by Chalak et al. [20], the IL-6 level at 6 and 72 h in the present study’s patients that had died, had severe disability, and had neuromotor retardation at month 12 was significantly higher than in those that were living without disability. In contrast to Chalak et al. [20], in the present study there wasn’t a correlation between the TNF-α level at 6 and 72 h, and adverse outcomes at month 12.

Roka et al. [8] performed a study to determine if TH causes a change in serum cytokine levels in newborns with HIE. They administered WBC in 10 newborns with moderate and severe HIE, but didn’t administer WBC in 8 newborns with moderate and severe HIE. The newborns with HIE that did and did not receive WBC were compared in terms of serum cytokine levels at 6, 12, and 24 h after birth. They reported that the IL-6 level at 6 h was significantly lower in the WBC group than in the non-WBC group. They suggested based on this result that TH rapidly suppresses cytokine production as a response to asphyxia and that this might be an important mechanism associated with the neuroprotective effect of TH. In the present study SHC or WBC was administered to each newborn with moderate and severe HIE, as withholding such treatment would have been unethical. Following TH, there weren’t any significant differences regarding change in the biomarker levels between the 2 methods. The IL-6 level at 6 h was significantly higher in the newborns with HIE (SHC, WBC, and mild HIE groups) than in the control group, which supports the notion that cytokines increase secondary to asphyxia. Moreover, there wasn’t a significant change in the IL-6 level from 6 to 72 h in the SHC or WBC groups, whereas the IL-6 level at 72 h was significantly lower than at 6 h in the mild HIE group even though TH wasn’t administered. This finding suggests that even if TH suppresses cytokine production, it can’t decrease the cytokine level to normal within 72 h of birth in newborns with moderate or severe HIE.

The most important limitation of the present study is the small study population; however, to the best of our knowledge this prospective, randomized single-center study is the first to compare the effects of SHC and WBC on biomarker levels in newborns with HIE. Another limitation is that biomarkers in CSF weren’t analyzed; had they been the findings might have differed. The biomarker levels were measured only in serum and urine samples, as they are more useful and their collection in less invasive.

## Conclusion

There weren’t any differences in the effects of SHC and WBC on the biomarkers evaluated in the present study. Serum IL-6 and protein S100 levels may be useful for diagnosing HIE, and the urine L/C ratio may be useful for differentiating newborns with moderate and severe HIE from those with mild HIE. The serum IL-6 level and the L/C ratio might also be useful for identifying cases of HIE that might result in disability or mortality. Additional larger scale and longer term studies might yield more generalizable findings.

## Abbreviations

aEEG: Amplitude integrated electroencephalography
CKBB: Brain-specific creatine kinase
CSF: Cerebrospinal fluid
HIE: Hypoxic ischemic encephalopathy
IL: Interleukin
L/C: Lactate/creatinine
NSE: Neuron spesific enolase
SHC: Selective head cooling
TH: Therapeutic hypothermia
TNF: Tumor necrosis factor
WBC: Whole body cooling
